# Asking questions that are “close to the bone”: integrating thematic analysis and natural language processing to explore the experiences of people with traumatic brain injuries engaging with patient-reported outcome measures

**DOI:** 10.3389/fdgth.2024.1387139

**Published:** 2024-06-25

**Authors:** Daniela Di Basilio, Lorraine King, Sarah Lloyd, Panayiotis Michael, Matthew Shardlow

**Affiliations:** ^1^ Division of Health Research, School of Health and Medicine, Lancaster University, Lancaster, United Kingdom; ^2^ Department of Neuropsychology, North Staffordshire Combined Healthcare NHS Trust, Stoke-on-Trent, United Kingdom; ^3^ Department of Psychology, Manchester Metropolitan University, Manchester, United Kingdom; ^4^ Department of Computing and Mathematics, Manchester Metropolitan University, Manchester, United Kingdom

**Keywords:** traumatic brain injury, patient-reported outcome measures, natural language processing, sentiment analysis, artificial intelligence, healthcare development

## Abstract

**Introduction:**

Patient-reported outcomes measures (PROMs) are valuable tools for assessing health-related quality of life and treatment effectiveness in individuals with traumatic brain injuries (TBIs). Understanding the experiences of individuals with TBIs in completing PROMs is crucial for improving their utility and relevance in clinical practice.

**Methods:**

Sixteen semi-structured interviews were conducted with a sample of individuals with TBIs. The interviews were transcribed verbatim and analysed using Thematic Analysis (TA) and Natural Language Processing (NLP) techniques to identify themes and emotional connotations related to the experiences of completing PROMs.

**Results:**

The TA of the data revealed six key themes regarding the experiences of individuals with TBIs in completing PROMs. Participants expressed varying levels of understanding and engagement with PROMs, with factors such as cognitive impairments and communication difficulties influencing their experiences. Additionally, insightful suggestions emerged on the barriers to the completion of PROMs, the factors facilitating it, and the suggestions for improving their contents and delivery methods. The sentiment analyses performed using NLP techniques allowed for the retrieval of the general sentimental and emotional “tones” in the participants’ narratives of their experiences with PROMs, which were mainly characterised by low positive sentiment connotations. Although mostly neutral, participants’ narratives also revealed the presence of emotions such as fear and, to a lesser extent, anger. The combination of a semantic and sentiment analysis of the experiences of people with TBIs rendered valuable information on the views and emotional responses to different aspects of the PROMs.

**Discussion:**

The findings highlighted the complexities involved in administering PROMs to individuals with TBIs and underscored the need for tailored approaches to accommodate their unique challenges. Integrating TA-based and NLP techniques can offer valuable insights into the experiences of individuals with TBIs and enhance the interpretation of qualitative data in this population.

## 1 Introduction

### 1.1 Traumatic brain injuries and the use of routine outcome measures to inform care decisions

The World Health Organization (WHO) defines Traumatic Brain Injuries (TBIs) as “an acute brain injury resulting from mechanical energy to the head from external physical forces”, excluding manifestations related to “drugs, alcohol, medications, caused by other injuries or treatment for other injuries (e.g., systemic injuries, facial injuries or intubation)”. It also excludes manifestations linked to “other problems (e.g., psychological trauma, language barriers or coexisting medical conditions) or caused by penetrating craniocerebral injury” (1). TBIs are a leading cause of death and disability worldwide, and their global incidence is rising (2) to the extent that TBIs are commonly referred to as “the silent epidemic” (3–5). Recent estimates (6) indicate that in 2019, there were 27.16 million new TBIs reported and a prevalence of 48.99 million TBIs worldwide. Nonetheless, exact figures on the incidence and prevalence of TBIs are difficult to retrieve due to missing data, methodological inconsistencies in epidemiological studies, out-of-hospital deaths, and lack of comprehensive, comparable and regularly updated epidemiological data (6–9).

Furthering our understanding of TBIs and their effects is essential in light of the remarkable impairment and severe limitations they can have on people's lives, particularly as TBIs are considered a life-long condition that affects individuals’ functioning and quality of life, as well as the wellbeing of their loved ones and society as a whole (10–14). In England and Wales, it has been calculated that ∼1.4 million patients per year attend hospital following head injury, and TBIs are the most common cause of death under the age of 40 years, with yearly costs estimated at €33 billion in Europe and £15 billion in the UK (0.8% of GDP) (15–17).

Given the intricate interplay between brain function and psychological health, assessing and evaluating mental health outcomes in individuals with TBIs becomes paramount for effective intervention and rehabilitation strategies (16, 18). A crucial role in guiding assessment and intervention is played by Patient-Reported Routine Outcome Measures (PROMs). PROMs are standardised tools and instruments designed to systematically assess and evaluate patients’ progress and outcomes of interventions over time (19). They are routinely used in standard care for TBI patients to assess the presence and intensity of common mental health conditions associated with TBIs, such as anxiety and depression, and inform therapeutic choices and evidence-based decision-making in clinical and research settings (20–23). In the UK, the national PROMs programme mandates that all hospitals utilise PROMs as part of healthcare interventions (24), with the aim of facilitating clinical decisions that are evidence-based, ensuring high care standards and promoting service development (25).

### 1.2 Key areas of evaluation via PROMs in individuals with TBIs

In the context of mental health in individuals with TBIs, PROMs have been repeatedly used in clinical and research practice to assess domains that are commonly negatively influenced by TBIs. These include, for example, global functioning, neuropsychological impairment, adjustment problems, and/or mood disturbances (26), with particular relevance given to the evaluation of anxiety and depression as common TBI comorbidities (12, 27, 28). A growing number of studies increasingly highlighted the importance of taking into account patients’ perspectives when assessing these domains and when planning and evaluating treatment outcomes (29–31).

Focusing on the experiences and views of patients when administering and scoring PROMs can serve different purposes, including supporting patients’ understanding of their symptoms, enhancing communication and treatment management and facilitating discharge planning (30, 32). Despite the advantages of PROMs, their implementation in the context of TBIs is not without challenges. Issues such as variability in injury severity, cognitive impairments, and the need for specialised measures present hurdles that may necessitate careful consideration and adaptation of PROMs (26, 33). More specifically, among others, barriers to completing PROMs for TBI patients include cognitive demands associated with PROMs completion, memory issues that can alter the possibility of accurately recalling symptoms, potentially impaired self-awareness, and cognitive biases (11, 34–36).

Additionally, literature on barriers and facilitators to PROMs completion identified both patient-related and professionals-related barriers. The former include issues with the contents of the PROMs, for example, if the questions are perceived as too generic and non-personal, or too complicated to be completed independently, without the help of healthcare professionals (30, 37, 38). Other studies reported that service users might be discouraged from completing PROMs if they feel that they have not received appropriate explanations as to their role and functions, or how the data collected will be used and safely stored (39–42). Language barriers and issues with PROMs questions perceived as ethically and/or culturally insensitive were also reported as factors potentially affecting PROMs completion rates (43, 44). Additionally, a plethora of studies also outlined professionals-related barriers to PROMs completion, including, for example, concerns about additional work and time commitment (e.g., need to be trained in PROMs administration and scoring), the perceived burden of having to educate patients on the value and uses of PROMs, and following-upon non-responders (8, 45–47).

In light of these considerations, the current study aimed at exploring the views, opinions and experiences of completing PROMs in a sample of individuals with TBIs referred to the Clinical Neuropsychology Department of the Salford Royal NHS Foundation Trust (SRFT), a large hospital located in the Northwest of England (UK).

## 2 Methods

### 2.1 Rationale

TBI patients referred to the SRFT Clinical Neuropsychology Department routinely complete a set of PROMs (for a complete list, see Table 1. below), both before their first appointment and again following discharge from the service. Patients receive the PROMs via post at their home address on both occasions, together with a pre-paid envelope to be able to return the PROMs once completed. An internal service evaluation conducted by Peak et al. (48) highlighted very low completion rates in the said department, with pre-intervention PROMs completed only by 30% of service users referred to the service, and post-intervention PROMs returned only by 7% of service users. As the authors (48) pointed out, these findings warranted further investigation into service users’ lived experiences of receiving and engaging with PROMs as part of their care. Therefore, the current study set out to:

- Explore the lived experiences of individuals with a TBI engaging with PROMs as part of their care pathway;
- Understand the factors acting as barriers and facilitators to the completion of pre-intervention and post-intervention PROMs;
- Evaluate potential challenges linked to ‘indirect aspects’ of PROMs that may affect completion (e.g., provision of paper-based copies, sent to service users via post).

**Table 1:** Description of PROMs used in the SRFT Clinical Neuropsychology Department.

Routine outcome measures (ROMs)	Characteristics and psychometric properties
Patient health questionnaire [PHQ-9; (49)]	The PHQ-9 measures the frequency of symptoms of depression using nine items on a 4-point Likert scale, ranging from 0 (not at all) to 3 (nearly every day). A total score comprised between 0 and 27 is obtained by summing all items; ordinary mean substitution is used for missing items if less than one-third (less than three items) are missing. Based on the total score of PHQ-9, the depression symptoms severity are categorised into minimal (0–4), mild (5–9), moderate (10–14), moderately severe (15–19), and severe (20–27) (49).
General anxiety disorder 7-item scale [GAD-7; (50)].	The GAD-7 is a brief self-report scale for symptoms of General Anxiety Disorder [GAD, (50)]. Seven items assess the frequency of anxiety symptoms on a 4-point Likert scale ranging from 0 (not at all) to 3 (nearly every day). A total score (min 0, max 21) is obtained by summing all items; ordinary mean substitution is used for missing items providing less than one third (less than two items) are missing. The total score is categorised into minimal (0–4), mild (5–9), moderate (10–14), and severe (15–21) anxiety symptoms (50).
Work and social adjustment scale [WSAS; (51)]	The WSAS is a five-item self-report scale of functional impairment assessing individual levels of functioning in everyday activities, including work, home management, family and relationship interaction and social and private leisure activities. Each of the five items is rated on a 9-point scale ranging from 0 (not at all a problem) to 8 (very severely impaired). The total scores range between 0 and 40, with high scores indicating higher levels of impairment.
TBI health checklist	The TBI health checklist is a measure developed ad hoc as part of the SRFT services provided to TBI patients taking part in this study. It assesses pre- and post-TBI conditions related to a variety of neurological and psychological domains, including neurological conditions (e.g., epileptic seizures), sensory difficulties (e.g., difficulty with vision and hearing), chronic pain and sleep disturbances. Respondents are asked to indicate the presence of such conditions (before and after reporting a TBI) using yes/no response options. A final open-ended question asks respondents about types of medication-if any-taken at the time of completion of this measure.

### 2.2 PROMs used to assess mental health and global adjustment in TBI patients

Table 1 below shows the PROMs routinely used to assess global functioning and TBI-related neurological and psychological symptoms in service users with a TBI accessing the SRFT Clinical Neuropsychology Department.

### 2.3 Participants

A purposive sampling method was used to recruit sixteen participants from the SRFT Clinical Neuropsychology Department. Full demographic information is purposedly not provided to protect participants’ anonymity. All participants had received a diagnosis of TBI and had been referred to the service to access psychological support to manage the effects of the TBI on their daily life (e.g., return to work) and/or to improve their mental health and wellbeing. A list of participants accessing the service was provided to the research team members, who utilised the following criteria to select service users to be invited to take part in the study: ≥18 years old; having mental capacity to consent to participation; having accessed the SRFT Clinical Neuropsychology services and having been sent copies of PROMs, although completion was not essential. This latter choice was due to the main aim of this study being to understand lived experiences of PROMs completion, including the barriers that may have hindered it.

Potential participants thus identified were contacted via phone call to inform them of the study and propose participation. If interested in participating, they were sent a copy of the Participant Information Sheet (PIS) and Consent Form (CF) via email or post, depending on their preferences. They were given a timeframe of at least one week to carefully consider participating in the study and allow them to ask any questions they may have had before participating. A suitable date and time were subsequently arranged for interviews to take place. Interviews were conducted until “theoretical saturation” was achieved (52–54), defined as “the point when no additional issues are identified and the codebook begins to stabilise” (52).

### 2.4 Data collection

Data for the current study were collected using semi-structured interviews conducted via phone calls, with each interview lasting about one hour. Interviews were audio recorded, and recordings were transcribed verbatim.

### 2.5 Ethics

The study received ethical approval from the SRFT Research & Innovation Department (approval n. S18HRANA45) and the Manchester Metropolitan University Research Ethics & Governance Committee (ns. 17158/9). All participants obtained written information (via PIS and CF) to be fully informed about the study and withdraw their participation. Audio-recorded consent was collected before the interviews and stored electronically, separately from the interview recordings, to ensure further protection of participants’ anonymity. Pseudonyms were assigned to participants at the start of each interview, and personal information was removed from the final copies of the transcripts. The study was conducted in agreement with the Code of Ethics of the Declaration of Helsinki (55).

### 2.6 Data analysis

The data for this study were analysed using different methods with the aim of capturing a wide range of semantic and structural features in the participants’ narratives. There is growing interest in the combination of more traditional analytical methods of qualitative data (e.g., thematic and interpretative phenomenological analysis) with innovative methods underpinned by machine learning (ML), such as Natural Language Processing (NLP) (56–59). Conventionally, the application of ML techniques, including NLP to text-based data, relied on large data sets for the algorithm to elicit meaningful themes. More recently, though, ML-based analysis of qualitative data yielded interesting results even on smaller data sets (60). This considered, the current study aimed to merge the potential of analysing data thematically and using NLP.

#### 2.6.1 Thematic analysis

The data collected were analysed utilising Thematic Analysis (TA), following Braun & Clarke's principles (61, 62). Codes and themes were identified inductively, using NVivo version 14 (63), a form of computer-assisted qualitative data analysis software (CAQDAS) that may help to advance the robustness of research findings (64). Investigator triangulation, defined as “the participation of two or more researchers in the same study to provide multiple observations and conclusions” (65), was used to enhance the methodological rigour and trustworthiness of the analytical process (66). To this aim, three research team members (DDB, SL and PM) analysed the corpus of data independently. Codes and themes were then collectively revised, and a final set of themes was agreed upon by consensus.

#### 2.6.2 Natural language processing

The term Natural Language Processing (NLP) refers to a subfield of computer science and artificial intelligence concerned with the automatic analysis, representation and understanding of human language (67). NLP typically focuses on written texts, processed and formatted for interpretation by a computer, such as the transcripts analysed in our research. Sub-fields of NLP include topic modelling (i.e., identifying common themes within a corpus of data by clustering words or sentences frequently appearing as connected) and sentiment analysis, which assigns meaning to words, phrases or other text units by matching them to their categorical sentiment (68–70). In recent years, NLP has been increasingly used in combination with qualitative methods of data analysis (56, 57, 71–73). The combination of these methods allows to achieve a textual and semantic analysis that integrates symbolic and statistical approaches (68, 74). Findings from studies utilising such a blending of researcher-driven qualitative analysis and NLP yielded valuable insight and complex interpretation of the data, showing the enhancing potential of combining these methods to analyse textual data (57, 59, 75, 76). Additionally, techniques that aim to predict the content -such as hate speech- or highlight the presence of emotional language in text-based data are routinely used in NLP to identify text characteristics that can subsequently be explored further within qualitative analysis (57, 77, 78). In light of this, integrating NLP with more traditional methods of qualitative data analysis could enhance the possibility for qualitative researchers to analyse larger amounts and more diverse data types, thus increasing the chance to appreciate the richness and complexity of human experience as portrayed by text-based data (79).

NLP tools make use of the same transformer-based large language models that are powering the current wave of interest in the AI field (80). The transformer model is a large neural network relying on the self-attention mechanism (81). This is particularly well suited for text analysis as the attention mechanism prioritises appropriate elements (e.g., words) of a sequence (e.g., a sentence) to make an overall classification (e.g., whether the text is positive or negative). The GPT architecture is best known for its use in conversational applications (82). In this configuration, a causal language modelling (CLM) objective is used to predict the next words forming a response to a user. However, in our case, we used Bert-based models (83) still relying on the transformer architecture using a Masked Language Modelling (MLM) objective. These models are particularly designed for the purpose of sequence classification, which was our goal.

We used two tools for our NLP analysis. Both of these tools take text as input (in this case, the sentences from the interviews). Prior to running our tools, we pre-processed the input text to separate the text into sentences and to distinguish between dialogue acts between the interviewer and the participant. We ran each tool separately on all sentences from the interviewer and the participant and aggregated the results using the mean average within each document. We used a pre-trained sentiment analysis tool based on the BERT architecture and an emotion recognition pipeline based on the RoBERTa architecture (83, 84). Both tools were downloaded from the HuggingFace repository and applied to our data corpus using an Apple MacBook M2 Pro with 16GB of RAM.

## 3 Results

### 3.1 Thematic analysis

**Figure 1:**
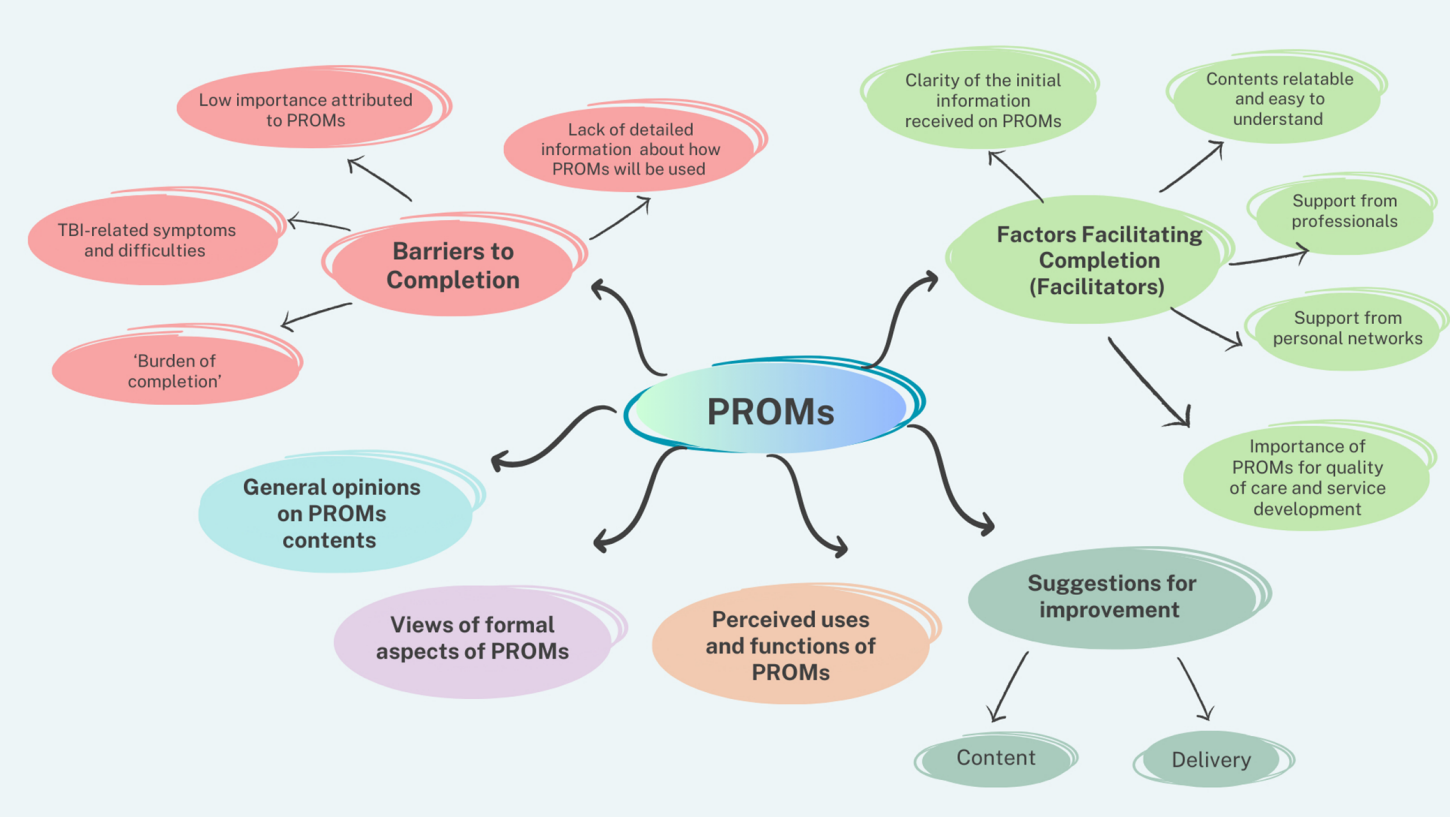
Map of themes identified using Thematic Analysis.

The process of inductive TA generated six overarching themes (displayed in Figure 1 and illustrated below with exemplary quotes), which captured different opinions and experiences of TBI service users receiving copies of the PROMs.

### 3.2 Theme 1: barriers to completion of PROMs

*Sub-themes:*

#### 3.2.1 Lack of detailed information about how PROMs will be used

One of the main barriers to completing PROMs reported by participants was related to the perceived lack of sufficient information regarding the purposes and potential outcomes of completing PROMs. Many highlighted the need for more precise explanations about how their responses would be utilised within the healthcare system. On this note, participant twelve noted:

*It doesn't really say why it's useful or what it's helpful (…). I think maybe you could do with putting a little more information for context (…) I think if (…) I understood why it was important and why it was helpful to more than just me (…)—it was helpful to others in general, I would have been more inclined to fill it in* (P12)

One of the service users who did not complete the PROMs commented that having more information on the PROMs would have made them feel more “in control”, thereby increasing their motivation to complete them.

*I like to feel in control of everything that's going on in a very uncontrolled time, um, I think it [having more information about the uses and relevance of PROMs] would solidify my desire to complete them, yeah* (P12)

Moreover, a lack of clear information about the uses and functions of PROMs might lead service users to draw incorrect inferences as to why PROMs are used, which in turn might represent a barrier to engaging with them:

*Other people will think oh their judging me or they want, they're rating me—you know-* (P7)

*‘Cause you just think it's another form to fill in. Nobody will… They'll just put it in a filing cabinet and it will be stored* (P11)

#### 3.2.2 Low importance attributed to completing PROMs

Some of the interviewees reported a lack of perceived relevance or benefit of completing PROMs, which, in some cases, represented a barrier to completion. This was particularly true for participants who had received support for their TBI-related difficulties and had completed their treatment journey, as participants ten and eleven explained:

*Especially if you've been discharged like once you've been discharged it becomes… It starts becoming a little bit like unimportant, cause you're sort of gone* (P10)

*From my point of view I didn't think, I didn't feel as if it was helping me, cause I've gone past all of that bit* (P11)

#### 3.2.3 Potentially upsetting questions

Some participants reported discomfort or distress when faced with questions that touched upon sensitive topics such as suicide, or the impact that their TBI-related difficulties had on their families and loved ones. In some cases, this emotional burden acted as a barrier to completing PROMs. More specifically, when asked about the questions that may discourage people from completing PROMs, service users often mentioned the ones related to suicidal thoughts and the feeling of disappointing loved ones due to TBI-related issues.

*I would say uhm (…) “that you've let your family down” is quite a hard one. (…) because I think even if you didn't think about letting your family down that question would definitely make you think about it (…) Which can be a little bit sad sometimes.* (P1)

Interestingly, however, the vast majority of service users reported that they felt it was important to ask these questions and to use straightforward language to do so, as they reflected relevant aspects of the experiences of people with TBIs. This was reported by some of the participants as follows.

*I know that they're the most important too you know it's funny in a way. (….) [and] No, I wouldn't ask them differently.* (P3)

*These things are always going to be a little bit close to the bone (…) If you ask anyone: “Oh have you felt like harming yourself”, do you know what I mean it's like a, I don't think there's any way you can ease someone into that sort of question* (P12)

*There have been times when those thoughts have been present, and having to answer that um question, honestly is quite hard (…) but I honestly don't know how you would ask it any other way either. Because it's an important piece of information that a practitioner needs to know, so yeah, I don't think, I think its worded as well as it can be* (P15)

#### 3.2.4 TBI-related symptoms and difficulties

Participants cited cognitive impairments resulting from their TBI as obstacles to accurately completing PROMs, with memory impairments and concentration problems being among the most frequently reported.

*The only problem I'd have is, if you ask a question, sort of before the accident, I can't remember, or after the accident—if you know what I mean* (P13)

*I know it's only nine questions but actually nine questions for some people especially seeing as question number seven is “do you have trouble concentrating on things such as reading the newspaper or watching television”, well actually some people would have trouble getting through nine questions* (P7)

*Over two weeks it's like, two weeks I can't even remember what happened (…) two days ago, never mind two weeks ago (…) at the time I wouldn't know if it would have been easy or not to fill it [the PROM] in. ‘Cause I can't even remember, you know, much about it really* (P9)

#### 3.2.5 “Burden of completion” (time and effort required to complete PROMs)

Some participants expressed frustration with the time-consuming nature of completing PROMs, particularly when dealing with physical or cognitive limitations that increased the effort required. Both participants eight and nine described this concept using the term “*overwhelming*” and participant ten added that the length and multiple iterations of the PROMs increased the burden of completion, thus representing a potential barrier.

*That just seems like a bit of a mare [Laughs]. A bit long. [Laughs]. (…) Uhm, if I was getting that very often then towards the end of it, I'd stop responding because it would get a bit tedious* (P10)

#### 3.2.6 Unclear how to best answer PROMs questions

Some participants struggled with understanding the intent behind specific questions or determining the most appropriate response, leading to uncertainty and dissatisfaction with the process and ultimately, to disengaging from PROMs.

*I generally just missed the questions that I don't really understand out (…) I would miss the ones that weren't specific enough or weren’t for me to answer because I don't have that kind of information.* (P10)

When asked whether not understanding how to answer could impact the likelihood of completing the PROMs, participant eight replied:

*It could do. I think I would still carry on, but I can see how with other people they might just think “yeah, I don't understand it, I can't do it”* (P8)

For some service users, the main difficulty laid in discerning the changes in their cognitive functions and mental health before and after the TBI, which affected their ability and willingness to engage with questions on such changes.

*It was quite difficult to differentiate between before my injury and after because everything on that list was impacted by my mental health prior to the events which led to my injury* (P15)

### 3.3 Theme 2: factors facilitating completion of PROMs

*Sub-themes:*

#### 3.3.1 Clarity of the initial information received on PROMs

Whilst some service users felt that the lack of clear information about the aims and uses of PROMs questions hindered their completion, the majority felt that receiving clear and comprehensive information about the purpose, relevance, and potential benefits of completing PROMs positively influenced their willingness to engage with these measures.

*I remember reading through it all and it was quite clear that it was important to do it uhm cause obviously it was for my benefit.* (P1)

One aspect that emerged as encouraging completion was receiving appropriate information on the ethical treatment of their data, including who would have access to their responses and how the confidentiality of their data would be protected.

*I know it's all in, um, kept secret anyway* (P13)

*It matters to me, cause confidentiality is you know, personal data, data protection.* (P13)

#### 3.3.2 PROMs contents perceived as relatable and easy to understand

Participants appreciated PROMs that used language and concepts they could easily grasp, enhancing their confidence in providing accurate responses. Furthermore, some of the interviewees reported being more inclined to complete PROMs if they felt that questions were appropriately capturing their symptomatology, rather than being too broad and generic.

*I think they're all quite (…) uhm not too difficult to answer, they're pretty straight forward and uh, I think it encompasses most of the things you feel when you've had an injury like this, I was quite happy with them* (P4)

Some of the interviewees also commented on the time ranges indicated on the PROMs being helpful in supporting a more accurate recall and evaluation of their symptoms.

*I found useful is where it says “over the past two weeks”, so it's giving an instruction, (…) not to think of things as a whole, but in that short space of time prior to completing it. Because I think that if it hadn't said that, I would've felt very overwhelmed* (P15)

#### 3.3.3 Importance of PROMs for quality of care and service development

Participants recognised the value of PROMs in improving the quality of care and advocating for their needs within the healthcare system, motivating them to complete these measures. In this regard, participant eleven highlighted the need to provide information on how PROMs can help to improve services, as a way to enhance completion rates.

*People need… (…) I think people need to know why they're doing it. (…) Because otherwise there doesn’t seem to be much point. And if it's too help them change stuff, people are more likely to fill them in* (P11)

The participants’ narratives also indicated that the motivation to complete PROMs could be increased by knowing the perceived benefits for oneself, other service users, and the service as a whole.

*It was quite clear that it was important to do it uhm cause obviously it was for my benefit* (P1)

*You'd fill it in because you think you're helping somebody else. And I think that's what most people think. Only because again (…) I don't want anybody else to go through what I've gone through* (P11)

#### 3.3.4 Support from caregivers and personal networks when completing PROMs

Participants emphasised the importance of emotional and practical support from family members, friends, or support networks in facilitating their completion of PROMs.

*My partner was here (…) when I first got the letter, and you know she (…) spoke throughout when I was completing it* (P4)

The same participant also remarked that caregivers of people with TBIs can not only support completion, but also offer valuable help to evaluate pre- and post-injury changes, which might be challenging to assess in case of memory issues.

*It's better to get somebody else's view who understands the situation and has seen how you've been really (…) it's better with another point of view as well, you know, to give the wider picture* (P4)

#### 3.3.5 Support from healthcare professionals

Some service users emphasised that encouragement, guidance, and assistance from healthcare professionals were instrumental in overcoming barriers to engaging with PROMs.

*I know for a fact that anything I've ever been given to or told by the doctors personally rather than the letters sent to my address, I'm more likely to take an automatic interest in* (P12)

*I find… It's best if I sit there and talk to the doctor. We have a good conversation she's a very good doctor* (P3)

Participant seven also mentioned the possibility of receiving support from non-clinical staff as a potential factor that would facilitate PROMs completion.

*Even like the reception staff could have some understanding of them and then if people need to ring up for clarification—you know they could ring and—you know-say: “look I've been given this to fill in but I don't understand this, is any chance you could explain it to me?”* (P7)

### 3.4 Theme 3: general opinions on PROMs contents

Participants provided varied feedback on the content of PROMs, with the vast majority expressing satisfaction with the comprehensiveness and relevance of the questions.

*It's quite hard to put into words how you're feeling and everything that you've listed sort of in those questions, at some point you will, you will feel like that. So I would say yeah, you've hit the nail in the head with all of them* (P1)

Some of the participants felt that the content of the questions proposed, although generally relatable, did not allow them to give sufficient voice to the subjective experience of living with a TBI.

*Umm everybody is different when they suffer from low mood and depression* (P4)

*Everybody's experience or feelings are different (…) It's hard to put into words because the feeling of it was, you're all individual (…) and I get they've got to group them as a whole to get everything together, but, there's no individuality. If that makes sense?* (P11)

Nonetheless, others had a clearer understanding of the PROMs as tools aiming to gather data which need to be comparable within and across services and service users, and therefore, need to entail validated questions capturing common TBI symptoms.

*If you want information analysing you’ve got to have a questionnaire that uhh… (…) perhaps addresses you know the most likely things that people are experiencing* (P2)

### 3.5 Theme 4: views of formal aspects of PROMs

Participants shared their perspectives on the formal aspects of PROMs, including the format, layout, terminology, and response options. Participant one commented on the layout of the questionnaires being clear, and their length being appropriate to gather sufficient information on different aspects of the physical and mental health of TBI patients.

*The layout's quite clear, so, you're not gonna get overwhelmed by you know loads and loads of questions* (P1)

When asked whether printing PROMs in colour would have influenced the motivation to complete them, participants unanimously indicated that it would not have made any significant difference:

*Um I think, colour doesn't massively matter to people but, I don't really think unless you're trying to get kids* (P12)

Some of the questions asked were also related to the scoring system (yes/no answers, or Likert scales) used in the measures composing the PROMs. Although there was some heterogeneity of views on the scale range (e.g., the GAD-7 four-point Likert scale) and labels (e.g., “several days”) of such measures, service users generally agreed that the response options and format were clear and easy to understand.

Lastly, a few participants pointed out that some of the terms used in the PROMs would benefit from more clarification, enabling respondents to understand their meaning better and provide more accurate answers. In this regard, participant seven remarked:

*I guess some of them, “other neurological condition” or “other mental health difficulties” (…) I guess that, the “other mental health difficulties” does that make it sound like a TBI is a mental health difficulty and you might have other mental health difficulties, other than you TBI? I don't know why the word “other” is there* (P7)

Participant eight echoed this point as follows:

*“Other neurological conditions” that… This may be confusing say if nobody's ever suffered a head injury before and know anything about it* (P8)

### 3.6 Theme 5: perceived uses and functions of PROMs for service provision

Participants discussed their perceptions of how PROMs could be used to improve service provision, including personalised care planning, monitoring treatment progress, and identifying areas for service development and improvement.

*You want to know who you're dealing with and what their problems are* (P5)

*I would imagine that it would be that um, the person that's receiving these-that's doing the consultation would be able to initially see maybe what course of treatment or action would be needed* (P15)

Participant nine felt that PROMs were mostly useful to healthcare professionals “*to see if you're doing a good job*” (P9)

When asked specifically about the perceived functions of the follow-up PROMs, sent after discharge from the service, participant seven commented:

*I guess to hopefully make sure that they feel that there has been an improvement in their [the service users’] condition through attending neuropsychology* (P7)

### 3.7 Theme 6: suggestions for future improvements

*Sub-themes:*

#### 3.7.1 Content improvement of PROMs

Some participants suggested revisions to the content of PROMs, mostly to capture a wider range of subjective experiences and symptoms associated with TBIs. However, an almost equal number of interviewees expressed awareness about the difficulty of appropriately adapting standardised measures (such as the ones composing PROMs) to capture the variety of challenges individuals with TBIs may face. In participant seven's words:

*You get such a broad range of patients with varying levels of difficulty, (…) do you change the entire system for the minority?* (P7)

Another participant also expressed scepticism on whether adding open-ended questions would improve the quality of the information retrieved via PROMs:

*Even if you would actually put like a big gap for people to write stuff, they won't write things. Because they just don't want to. They're just not in the mood to do it. (…) Because then they've got to start thinking. And they've got other things to think about* (P11)

A smaller number of participants suggested minor changes in the contents of the questions, to make them more accurate and informative. For example, participant two suggested:

*The fifth question, “because of my injury my ability to form and maintain close relationships with others”. I'm not quite sure about that question you know (…) to receive this question a few weeks after a traumatic brain injury, you aren't really going to be able to assess that. Uhm perhaps it would be more relevant to (…) “the way I think about the relationships with my loved ones or those I live with has changed”* (P2)

#### 3.7.2 Improving the delivery of PROMs

Recommendations for enhancing the delivery of PROMs included providing additional support and guidance to patients and offering alternative formats and platforms for completion. This last aspect—the need to provide a range of different formats for PROMs- emerged in many of the service users’ interviews.

*I know for a fact if it was me and I got a letter through saying “you can either fill this out, get a phone call or go and have an appointment”, I probably would have done the phone call to be honest. (…) So yeah, just give people the option really I would say* (P1)

*In a different um, service, using similar PROMs type questionnaires, (…) I was actually given an iPad before my first appointment when I got there, to complete it and then I was asked at the end of my last appointment if I would stay behind for ten minutes* (P15)

*A large majority of us are online now (…) and communicate by email, and you could save up a lot of money a lot of paper and a lot of you know (…) by just simply sending out the information in an email* (P2)

A subgroup of interviewees indicated that the current way of sending PROMs (in paper copies, sent via post) might be the most appropriate for people with TBIs, who may struggle with engaging with PROMs delivered digitally (e.g., via a web link or email).

*I think post… Something as comprehensive as these the post is, is a good system. Email sounds… Makes it easier for the sender, it doesn't necessarily make it easier for the receiver.* (P5)

*I just forget things me because of my memory, I just forget, so yeah, post them and then, anything that comes through to the house, you know (…) NHS is opened* (P6)

Lastly, Participant fifteen suggested the option to ask people to complete PROMs in clinical settings to increase the availability of support and, in turn, completion rates.

*If somebody does feel they need that further input (…) potentially, setting somewhere aside for them to do that within the clinic setting* (P15)

### 3.8 Sentiment and emotion analysis

**Figure 2:**
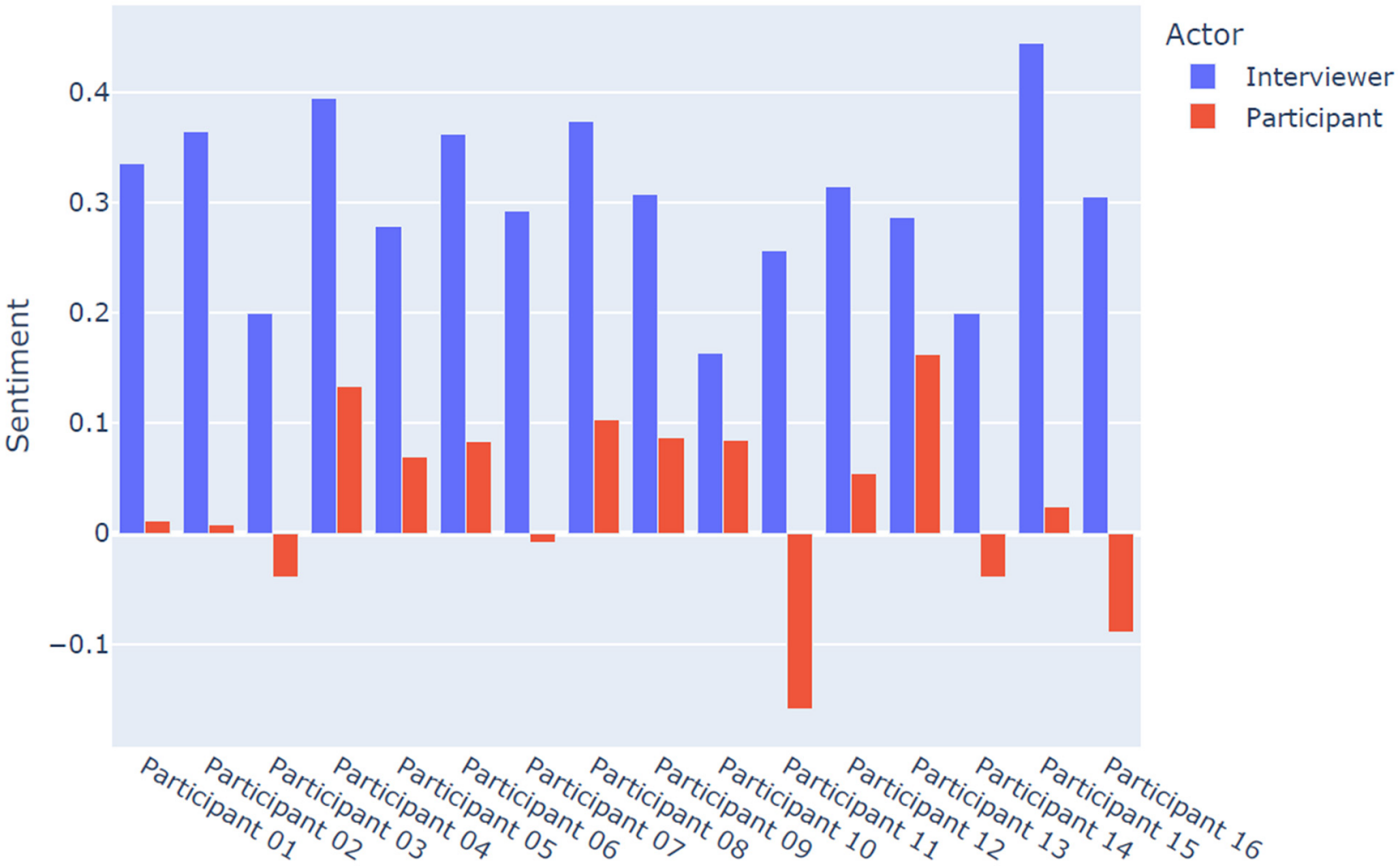
Sentiment analysis of participants and interviewers.

The sentiment and emotion analysis of the corpus of data rendered interesting findings related to the emotional connotations characterising the interviewers’ and service users’ discourses on engaging with PROMs. An overview of the findings for each interview is reported in Figure 2 below.

Figure 2 shows the results related to the polarity classification of sentences as positive or negative based on patterns of sentiment learnt from previous examples, as expressed by interviewers and participants (service users) taking part in the interviews. The results suggested that the sentiments expressed by interviewers were positive, with some variations of intensity across interviews. More heterogeneous, instead, were the sentiment connotations related to service users, with most of the interviews showing low levels of positive sentiments. Some of these interviews (i.e., participants three, seven, eleven, fourteen and sixteen) also showed a clear negative polarisation in the overall sentiment underpinning the participants’ narratives. Although the interviews addressed the service users’ experiences of engaging with PROMs, the low levels of positive sentiment and the presence of negative sentiment connotations might at least partly be due to the emotional challenges linked to discussing TBI-related challenges and possible changes in health-related quality of life.

**Figure 3:**
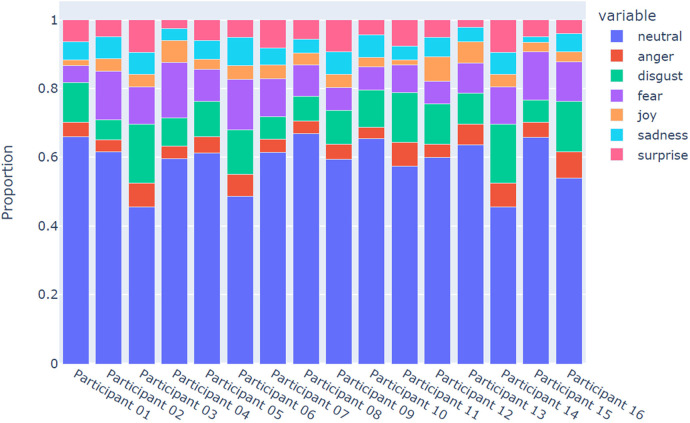
Emotional analysis of participants’ narratives.

Figure 3 below offers a more in-depth breakdown of the emotions retrieved via the NLP analysis of the participants’ experiences.

The majority of patient-reported experiences of engaging with PROMs were predominantly “neutral”. However, emotions of fear, disgust, anger and sadness also seemed to emerge frequently across all interviews. In this context, and in line with other studies using NLP techniques to analyse text-based data (85, 86), “disgust” is to be intended as “venting out”, expressing criticism and displeasure/complaint, rather than in a literal sense as “being disgusted”. More positive emotions (e.g., surprise and joy) were also present, albeit to a lesser extent.

## 4 Discussion

The current study explored the lived experiences and opinions of TBI service users receiving PROMs questionnaires as part of their routine healthcare. The sentiment analysis of the participants’ narratives about their engagement with PROMs and, more generally, about living with a TBI, revealed a generally low positive emotional tone in their narratives, with some participants expressing more polarised negative feelings. Further analysis of the emotions expressed during the interviews highlighted an overall “neutral” emotional tone. Interestingly, however, service users’ narratives also showed a connotation of negative emotions such as fear, disgust/disappointment and anger. Taken together, these findings call for the need to pay closer attention to the “emotional experiences” of TBI patients engaging with PROMs, as they may play a pivotal role in understanding how to improve their experiences with outcome measures and, in turn, increase completion rates.

The TA of participants’ narratives suggested that overall, people with TBIs had positive experiences of completing these measures and found them generally clear and easy to engage with. These positive views also encompassed aspects such as the layout and response format of the questionnaires composing the PROMs. Nonetheless, a few participants pointed out that some of the response options (e.g., “most days”) and questions asked (e.g., presence of chronic pain and “other general neurological conditions”) would have benefitted from further clarification, to allow for more appropriate answers and a more user-friendly experience of engaging with these measures.

The findings also highlighted a set of barriers to the completion of PROMs that ought to be considered by clinicians and policymakers. Among the barriers mentioned by participants, there were aspects related to the difficulty in completing PROMs and the need for more information on their role and importance for service provision and improvement. The difficulties affecting completion were mostly related to TBI symptoms, such as memory impairment and response fatigue, which are commonly reported by individuals with TBIs (87–89). This calls for the need to keep PROMs conceptually relevant but also accessible and easy to understand, particularly in consideration of the cognitive challenges that might create an additional burden to people with a TBI engaging with self-reported outcome measures (90, 91). Strategies to address these barriers may include, for example, providing clear instructions on the aims and uses of PROMs and using accessible formats.

Conversely, participants also identified factors that facilitated their engagement with PROMs. These included personalised support from caregivers and healthcare providers, PROMs questions perceived as relatable and easy to understand and high importance given to PROMs to improve their overall treatment and recovery process, as well as for service provision and development. Literature on TBIs previously highlighted the importance of caregivers’ support in different aspects of people's lives (92–94), and the findings from this study indicated that caregivers (close friends and family members) can play a pivotal role in influencing the completion of PROMs. Therefore, it appears to be essential for services to facilitate the involvement of service users’ networks when administering these measures. This is in line with the findings of a recent review (95) suggesting that caregiver-led support can prove to be useful in managing TBI-related difficulties, including the ones (e.g., cognitive deficits) that the current study identified as potential barriers to PROMs completion. Nonetheless, it is also crucial to recognise that the involvement of caregivers during PROMs completion would need to be limited and, where possible, monitored by services and professionals, as the administration of PROMs by proxies has the potential to introduce bias (96). Some participants pointed out that a potential facilitator to PROMs completion would be receiving support from healthcare professionals. If implemented, this type of support might call for the need for specific training, as professionals’ level of knowledge and confidence about using outcome measures can deeply influence their attitudes towards PROMs, which might, in turn, influence the quality of support offered (30, 97).

A further factor enhancing service users’ likelihood to engage with PROMs was the perception that the data collected would have been handled according to ethical regulations (e.g., safe storage and protection of patient identity) and used to inform care pathways and service development. This is coherent with previous literature on the barriers and facilitators to PROMs completion in different clinical populations (98, 99), indicating that when patients perceive PROMs as key for service quality and improvement, they are likely to be more motivated to engage with them.

Lastly, service users reported valuable suggestions for improving PROMs, such as incorporating open-ended questions to capture nuanced experiences, allowing for customisation of PROMs based on individual preferences and needs, and integrating technology-based solutions (e.g., using tablets) to enhance accessibility and user experience. Implementing these suggestions could enhance the relevance and utility of PROMs in clinical practice.

Our study combined TA and NLP analysis, incorporating sentiment analysis and emotion analysis. We were able to identify common themes in both analyses, and both analyses demonstrated that participants experienced distress during the interviews. The TA can provide context to this, suggesting that the questions asked by the interviewers (particularly around risk and the impact of TBI-related changes on families) can be a cause of distress. The NLP analysis allows us to quantify these factors for each participant in the study. In the future, the combination of TA and automated analysis could be used to give quantified yet meaningful insights into participant experiences.

Lastly, it was interesting to note that the sentiment of the interviewers was more positive than that of the participants. This finding is at least partly motivated by the fact that interviewers were acting in a professional capacity whilst participants were discussing their lived experiences entailing emotionally loaded topics (as demonstrated by the emotion analysis). Nonetheless, the experiences of both these groups, reflected in the positive and negative sentiments evidenced, provide valuable insights into the “positionality” of these “actors” (interviewer and interviewee). Further research combining TA and NLP should focus on the mutual links, if any, between the affective and emotional connotations expressed by the two “actors” in an interview setting, as this could inform “best practices” on how to conduct interviews addressing sensitive topics with people with complex clinical conditions, including but not limited to TBIs.

In summary, our study contributes to a deeper understanding of the lived experiences of individuals with TBIs completing PROMs, highlighting both the challenges and opportunities for optimising their use in clinical practice. Future research should focus on implementing participant-centred approaches to PROMs development and evaluation, as well as evaluating the impact that the emotional experiences of engaging with PROMs might have on their completion rates.

## 5 Limitations

Despite the promising benefits of combining NLP and traditional methods of qualitative data analysis (72), it is important to recognise that there are still some challenges related to the joint application of these methods. For example, there is a longstanding tradition of tools and strategies used within the qualitative research domain to ensure the rigour and trustworthiness of the themes retrieved and interpretations provided. These include, among others, triangulation, peer review or debriefing, member-checking, and external audits (the process by which researchers get audited by external researchers) (100, 101). Although these methods of ensuring quality are not entirely exempt from criticism (102–105), it is undeniable that similar debates on the rigour, trustworthiness and replicability of data analysis performed via NLP are still in their infancy (106, 107). In this regard, emerging debates highlight that not all NLP tools and methods that are consensually used have gone through systematic and comprehensive standardisation processes to ensure their validation and integrity (108, 109). In light of this, one of the salient challenges of the growing application of NLP methods in the research context will be to ensure a rigorous evaluation and optimisation of data analysis processes and transparency in the way results are obtained and explained (110). Being an integral part of these debates will allow qualitative researchers to contribute to shaping the so-called “computational turn”, i.e., the process by which new techniques and methodologies drawn from computer science (including but not limited to NLP) are implemented in the humanities and social sciences (109).

Future research combining NLP and more conventional qualitative data analysis methods will need to strive to achieve “a delicate balance between capitalising on digital advantages and upholding research integrity” (109, p. 577). As for any other research paradigm, one of the main goals of mixed-methods research integrating NLP methods will be to abide by high methodological standards, minimising the risk of bias and maximising the accuracy and credibility of research results (111). To do so, researchers will need to strive for rigour by adopting a coherent application of analytical methods and clear standards of honesty and transparency in reporting the study results (112, 113).

## Data Availability

The datasets presented in this article are not readily available because of the need to protect participants’ anonymity and the confidentiality of their data, as the qualitative nature of the dataset might expose them to the risk of being identified. Requests to access the datasets should be directed to d.dibasilio@lancaster.ac.uk.
