# Supplementary material for: Asking questions that are “close to the bone”: integrating thematic analysis and natural language processing to explore the experiences of people with traumatic brain injuries engaging with patient-reported outcome measures
Source: Front Digit Health. 2024 Jun 25;6:1387139. doi: 10.3389/fdgth.2024.1387139 (PMC11231399; doi:10.3389/fdgth.2024.1387139)
Supplement: Supplementary file 1 [file Table1.docx]

Supplementary Material

**Table 1. *Description of ROMs used in the Clinical Neuropsychology Department (Name of the Hospital masked for Blind Review)***

| **Routine Outcome Measures (ROMs)** | **Characteristics and Psychometric Properties** |
| --- | --- |
| **Patient Health Questionnaire** (PHQ-9; (Kroenke et al., 2001) | The PHQ-9 measures the frequency of symptoms of depression using nine items on a 4-point Likert scale, ranging from 0 (not at all) to 3 (nearly every day). A total score comprised between 0 and 27 is obtained by summing all items; ordinary mean substitution is used for missing items if less than one-third (less than three items) are missing. Based on the total score of PHQ-9, the depression symptoms severity are categorised into minimal (0–4), mild (5–9), moderate (10–14), moderately severe (15–19), and severe (20–27) (Kroenke et al., 2001). |
| **General Anxiety Disorder 7-item scale (**GAD-7; Spitzer et al., 2006). | The GAD-7 is a brief self-report scale for symptoms of General Anxiety Disorder (GAD, Spitzer et al., 2006). Seven items assess the frequency of anxiety symptoms on a 4-point Likert scale ranging from 0 (not at all) to 3 (nearly every day). A total score (min 0, max 21) is obtained by summing all items; ordinary mean substitution is used for missing items providing less than one third (less than two items) are missing. The total score is categorised into minimal (0–4), mild (5–9), moderate (10–14), and severe (15–21) anxiety symptoms (Spitzer et al., 2006). |
| **Work and Social Adjustment Scale** (WSAS; Marks, 1986) | The WSAS is a five-item self-report scale of functional impairment assessing individual levels of functioning in everyday activities, including work, home management, family and relationship interaction and social and private leisure activities. Each of the five items is rated on a 9-point scale ranging from 0 (not at all a problem) to 8 (very severely impaired). The total scores range between 0 and 40, with high scores indicating higher levels of impairment. |
| **TBI Health Checklist** | The TBI health checklist is a measure developed ad-hoc as part of the services provided by the health service accessed by TBI patients taking part in this study. It assesses pre- and post-TBI conditions related to a variety of neurological and psychological domains, including neurological conditions (e.g., epileptic seizures), sensory difficulties (e.g., difficulty with vision and hearing), chronic pain and sleep disturbances. Respondents are asked to indicate the presence of such conditions (before and after reporting a TBI) using yes/no response options. A final open-ended question asks respondents about types of medication -if any- taken at the time of completion of this measure. |

**Table 2. Sentiment Analysis of Participants** **and Interviewers**

**
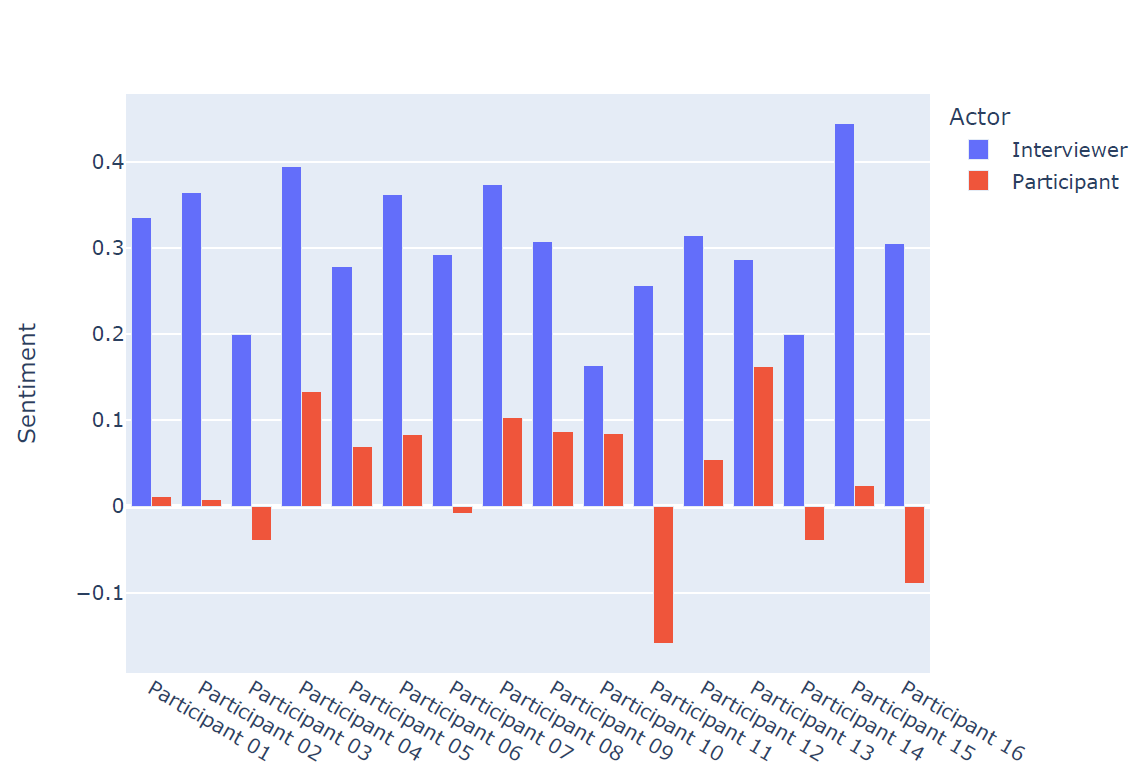
**

**Table 3. Emotional Analysis of Participants’ Narratives**

**
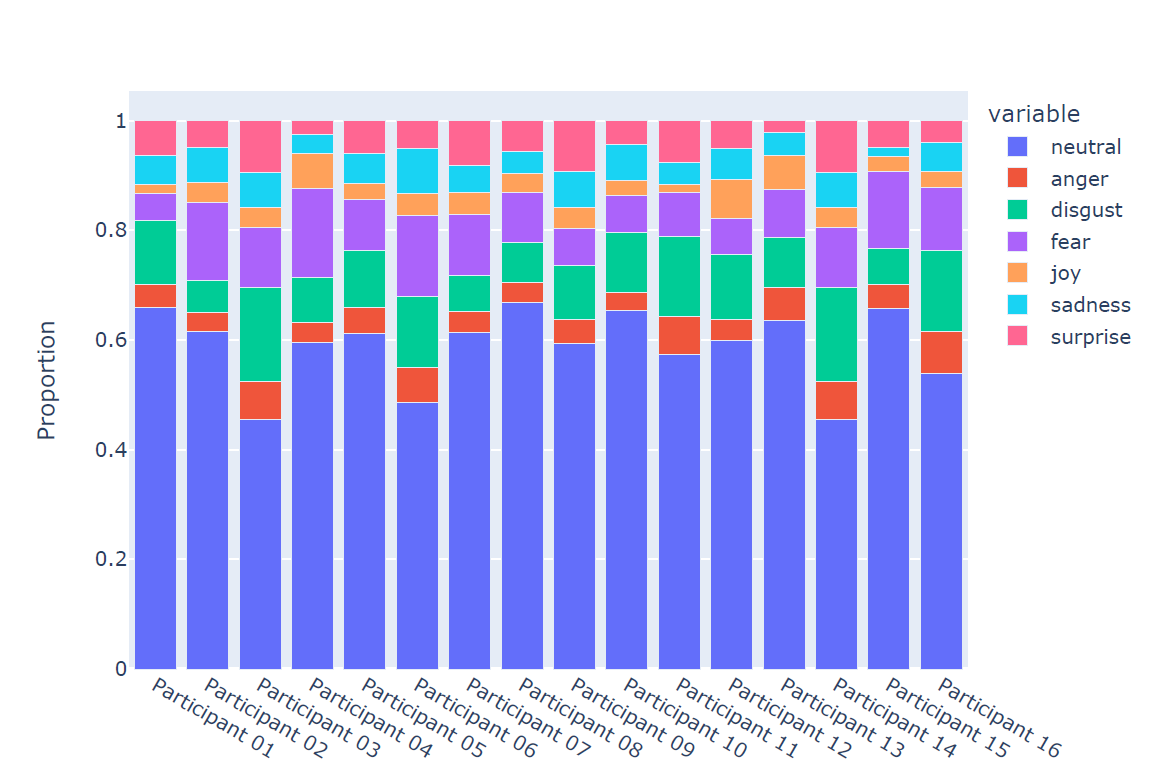
**
